# Free‐running 3D whole heart myocardial T_1_ mapping with isotropic spatial resolution

**DOI:** 10.1002/mrm.27811

**Published:** 2019-05-17

**Authors:** Haikun Qi, Olivier Jaubert, Aurelien Bustin, Gastao Cruz, Huijun Chen, René Botnar, Claudia Prieto

**Affiliations:** ^1^ School of Biomedical Engineering and Imaging Sciences King's College London London United Kingdom; ^2^ Center for Biomedical Imaging Research, Department of Biomedical Engineering Tsinghua University Beijing China; ^3^ Escuela de Ingeniería Pontificia Universidad Católica de Chile Santiago Chile

**Keywords:** 3D radial, free‐running, inversion recovery, myocardial T_1_ mapping

## Abstract

**Purpose:**

To develop a free‐running (free‐breathing, retrospective cardiac gating) 3D myocardial T_1_ mapping with isotropic spatial resolution.

**Methods:**

The free‐running sequence is inversion recovery (IR)‐prepared followed by continuous 3D golden angle radial data acquisition. 1D respiratory motion signal is extracted from the k‐space center of all spokes and used to bin the k‐space data into different respiratory states, enabling estimation and correction of 3D translational respiratory motion, whereas cardiac motion is recorded using electrocardiography and synchronized with data acquisition. 3D translational respiratory motion compensated T_1_ maps at diastole and systole were generated with 1.5 mm isotropic spatial resolution with low‐rank inversion and high‐dimensionality patch‐based undersampled reconstruction. The technique was validated against conventional methods in phantom and 9 healthy subjects.

**Results:**

Phantom results demonstrated good agreement (R^2 ^= 0.99) of T_1_ estimation with reference method. Homogeneous systolic and diastolic 3D T_1_ maps were reconstructed from the proposed technique. Diastolic septal T_1_ estimated with the proposed method (1140 ± 36 ms) was comparable to the saturation recovery single‐shot acquisition (SASHA) sequence (1153 ± 49 ms), but was higher than the modified Look‐Locker inversion recovery (MOLLI) sequence (1037 ± 33 ms). Precision of the proposed method (42 ± 8 ms) was comparable to MOLLI (41 ± 7 ms) and improved with respect to SASHA (87 ± 19 ms).

**Conclusions:**

The proposed free‐running whole heart T_1_ mapping method allows for reconstruction of isotropic resolution 3D T_1_ maps at different cardiac phases, serving as a promising tool for whole heart myocardial tissue characterization.

## 1 INTRODUCTION

Cardiovascular magnetic resonance has been increasingly used to diagnose and monitor different cardiac diseases.^1^, ^2^ Quantitative mapping of tissue parameters has demonstrated the potential to characterize subtle pathological changes of the myocardium and is especially valuable in detecting both focal and diffuse fibrosis.^3^, ^4^ T_1_ is sensitive to the changes of myocardial tissue properties, such as fibrosis, fat, and water content, and therefore is considered a promising novel biomarker to characterize myocardium and assess various cardiomyopathies.^5^, ^6^, ^7^, ^8^ In addition, extracellular volume fraction, which is important to detect diffuse myocardial fibrosis, can be estimated from native and post‐contrast myocardial T_1_ mapping.^9^ Myocardial T_1_ mapping has been recognized as one of the most valuable quantitative mapping techniques to support diagnostic, therapeutic, and prognostic decision making in ischemic and non‐ischemic cardiomyopathies.^4^

The modified Look‐Locker inversion recovery (MOLLI)^10^ and saturation recovery single‐shot acquisition (SASHA)^11^ are commonly used cardiac T_1_ mapping techniques. They acquire multiple images with different T_1_ contrasts and estimate pixel‐wise T_1_ by fitting the acquired signal to an exponential recovery model. Respiratory and cardiac motion may result in blurring artefacts in each T_1_‐weighted image and can cause misalignment between different images, influencing the T_1_ mapping accuracy.^12^ Therefore, breath‐holding or respiratory gating and electrocardiography (ECG) triggering are usually required during cardiac imaging. However, besides the requirement of patient compliance, a single breath‐hold limits acquisition time and results in low spatial resolution and limited coverage as typically only 1 image is acquired per cardiac cycle.^10^, ^11^ Multiple breath‐holds may allow longer acquisition time, but may result in different breath‐hold positions, which can lead to mis‐registration artefacts and inaccuracy of T_1_ estimation.^13^ Respiratory gating has been used to limit respiratory motion during free‐breathing imaging, but has low imaging efficiency and may lead to unpredictable long acquisition time.^14^ ECG triggering is usually used to gate the acquisition only at diastole in each cardiac cycle, which has low acquisition efficiency and can be challenging in arrhythmic patients who have shortened diastolic phase.

Recently, the magnetic resonance multitasking technique using 2D radial acquisition has been proposed for motion‐resolved quantitative myocardial T_1_ and T_2_ imaging without breath‐holding or ECG triggering.^15^, ^16^ 2D myocardial mapping is typically performed with thick slices to avoid the influence of through‐plane motion^13^ and low SNR. 3D acquisitions can overcome these limitations and provide whole heart coverage for comprehensive characterization of diffuse diseases of myocardium.^17^ However, 3D T_1_ mapping is technically challenging, because respiratory gating and ECG‐triggering together with the requirement of whole heart coverage, may lead to very long scan time. To cover the whole heart in an acceptable scan duration, current 3D T_1_ mapping techniques^14^, ^18^ acquire only a few slices in the short‐axis view with a slice thickness of >8 mm, which require complex scan planning and may suffer from partial volume effect along the slice direction.

To address the current technical challenges of myocardial T_1_ mapping, we propose a free‐running (free‐breathing, retrospective cardiac gating) 3D whole heart T_1_ mapping technique with high isotropic spatial resolution (1.5 mm^3^), which is able to provide images in any desired orientation. The proposed sequence consists of an inversion recovery (IR)‐prepulse, followed by an efficient 3D golden angle radial acquisition^19^ to sample the T_1_ relaxation curve. The k‐space center is repeatedly sampled within each spoke and used to estimate 1D respiratory motion. ECG signal is recorded and synchronized with data acquisition. Respiratory binning is performed based on the estimated 1D respiratory signal, enabling estimation and correction of 3D translational respiratory motion. For the reconstruction, a recently proposed algorithm that combines dictionary‐based low‐rank inversion^20^ and high‐dimensionality 3D patch‐based undersampled reconstruction (HD‐PROST)^21^ is used. The proposed approach allows for respiratory motion compensated 3D myocardial T_1_ mapping at different cardiac phases. The feasibility, accuracy and precision of the proposed technique was validated against conventional T_1_ mapping methods in a standardized T_1_ phantom and 9 healthy subjects.

## 2 METHODS

### 2.1 Free‐running 3D T_1_ mapping sequence

**Figure 1:**
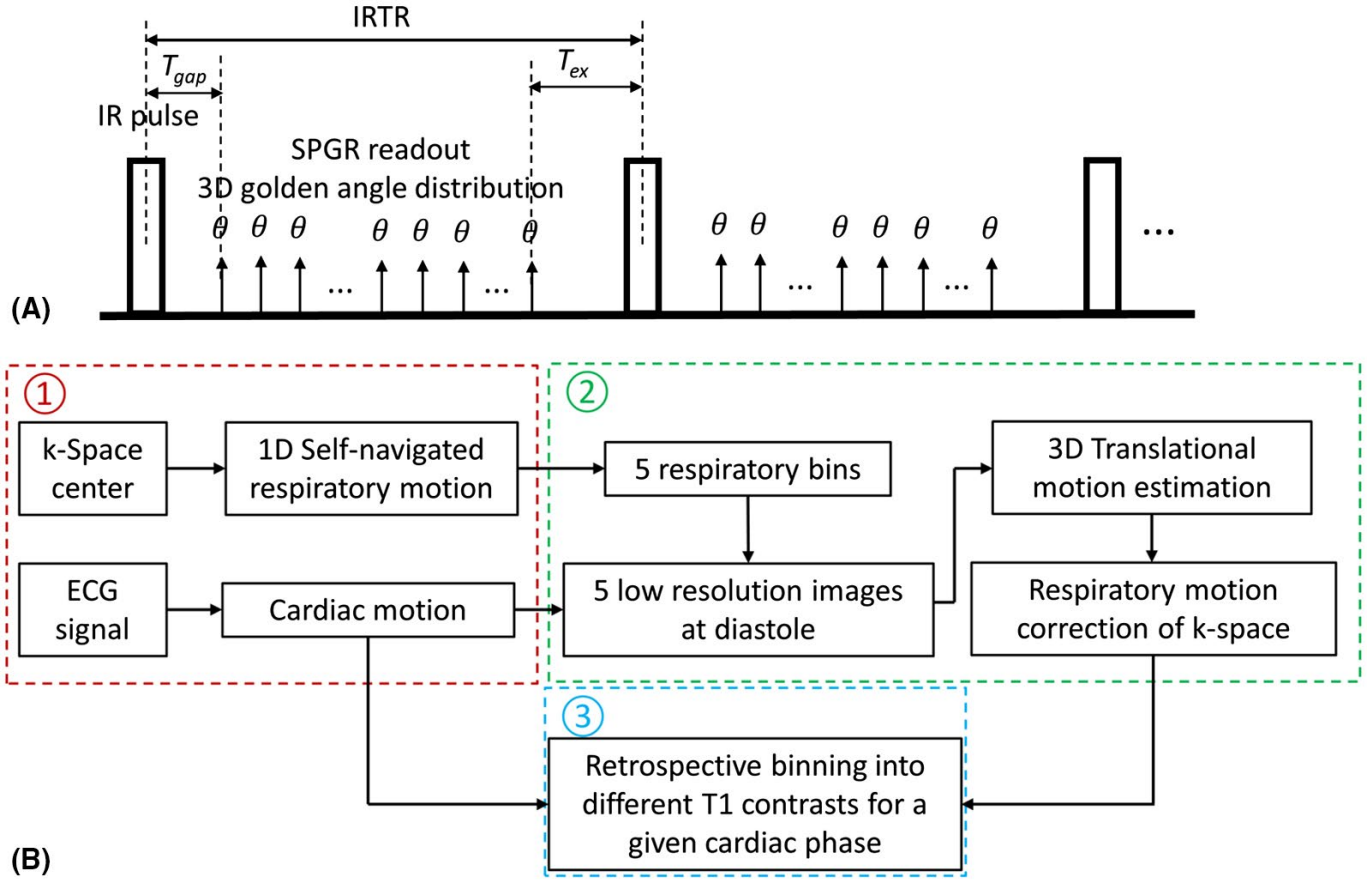
(A) Schematic diagram of the proposed free‐running myocardial 3D T_1_ mapping sequence. After inversion recovery (IR) preparation, spoiled gradient echo (SPGR) readout with low flip angle (θ) was performed using 3D golden angle radial trajectory. T_gap_ is the time between the IR pulse and the first excitation and T_ex_ is the time interval between the last excitation in the SPGR readout and the next IR pulse. IRTR is the IR repetition time. (B) Data sorting process for reconstruction of multiple T_1_ contrasts for a given cardiac phase, which includes 3 steps: 1D respiratory motion estimation from k‐space center of all radial spokes and cardiac motion extraction from ECG log; respiratory motion correction of k‐space using motion parameters estimated by 3D translational image registration of respiratory bin images at diastole; binning the respiratory motion corrected k‐space into different T_1_ contrasts for a given cardiac phase

The proposed IR‐prepared 3D radial sequence is shown in Figure 1A. IR‐preparation is used to generate T_1_ contrast, after which a spoiled gradient‐echo (SPGR) readout is performed with small flip angle (θ). Spatial‐spectral pulse for selective water excitation is adopted for fat suppression. To achieve pseudo‐uniform distribution of radial spokes for retrospective cardiac and respiratory binning, the continuously acquired radial spokes conform to the 3D golden angle distribution.^19^ Specifically, the azimuthal angle and polar angle increments (Δυ, Δτ) between adjacent spokes are defined as:

(1) $$\Delta \upsilon =\text{acos}\left(\phi_{1}\right),\;\Delta \tau =2\pi \;\cdot \;\phi_{2},$$

where *ϕ*_1_ = 0.4656, *ϕ*_2_ = 0.6823 are the 2D golden angle means.^19^

### 2.2 Motion extraction and retrospective sorting

**Figure 2:**
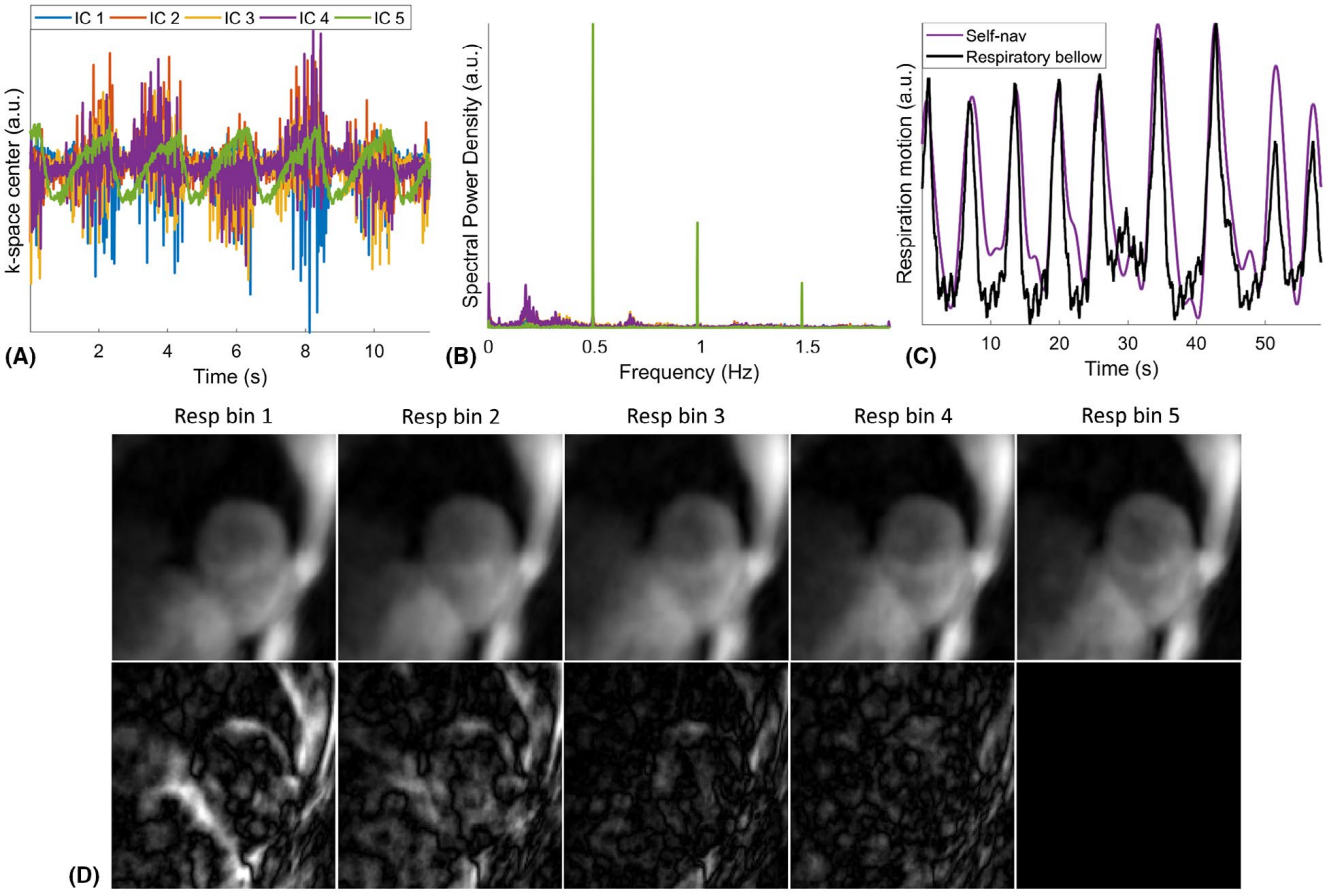
(A) The 5 independent components (IC) extracted from the k‐space center of all spokes using independent component analysis. (B) The spectral power of the signal components in (A). (C) The IC (IC 4) with highest spectral power in the respiratory frequency range (0.1–0.5 Hz) is selected and band‐pass filtered and compared with respiration bellow. (D) Example of low‐resolution self‐navigated images of 5 respiratory bins from intermediate reconstruction for 3D translational respiratory motion estimation. Setting bin 5 as the reference, its difference with other respiratory bins are shown in the second row to demonstrate the extent of respiration‐induced motion of the heart in this subject. The difference images are demonstrated with the same gray level range

The flowchart of retrospective processing, including motion extraction, respiratory motion correction and data sorting for T_1_ mapping of a given cardiac phase is shown in Figure 1B. Independent component analysis (ICA) was performed on the k‐space center of all spokes from all coils to extract the signal component that is most relevant to the 1D superior‐inferior respiratory motion. This approach has been previously validated for cardiac cine and coronary artery MRI using either radial or spiral trajectory.^22^, ^23^ To extract the self‐navigated respiratory signal from the k‐space center, coil compression is first performed using principle components analysis. ICA is applied to the k‐space center amplitudes from the compressed coils to obtain 5 independent components (IC). Example of the extracted ICs and the corresponding spectral power are shown in Figure 2A and B. The harmonic corresponding to the contrast change because of IR preparation is removed from the ICs based on the IR repetition time (IRTR in Figure 1A). Then, the IC with the highest spectral power in the respiratory frequency range (0.1–0.5 Hz) is selected (IC 4 in Figure 2A) and band‐pass filtered to get the 1D respiratory signal, based on which the k‐space data is binned into 5 equally populated respiratory phases from end‐inspiration to end‐expiration. An example of the estimated respiratory signal validated against the corresponding respiratory bellow signal is shown in Figure 2C. Cardiac motion synchronization is achieved by logging the ECG time stamps, from which the temporal occurrence of each spoke relative to the most recent cardiac R‐wave preceding the spoke is calculated and termed as cardiac delay.

After retrospective respiratory binning and cardiac gating, 3D translational respiratory motion is estimated via registration from the intermediate reconstruction of low‐resolution respiratory bin images at diastolic cardiac phase by using central part of selected k‐space data. Representative diastolic bin images of 5 respiratory motion states and the difference images with the end‐expiratory bin are shown in Figure 2D. A rectangular volume of interest for motion estimation is selected around the heart at the end‐expiratory bin and propagated to other respiratory bins. Respiratory motion correction is performed by correcting the phase of the k‐space data using the estimated 3D translational motion parameters. The respiratory motion corrected k‐space data can then be binned according to the cardiac delay and inversion recovery time (TI) for T_1_ mapping at a given cardiac phase.

### 2.3 Image reconstruction

#### 2.3.1 HD‐PROST algorithm

For 3D T_1_ mapping at a given cardiac phase, spokes for the corresponding cardiac delay and acquisition window are selected, which are then sorted into a time series of T_1_ contrasts according to TI and a given temporal window for each T_1_ frame. After data selection, the highly undersampled 3D radial data is reconstructed over multiple T_1_ contrasts by combining a dictionary‐based low‐rank inversion^20^ to efficiently reduce the number of T_1_ contrasts to be reconstructed with a recently proposed high‐dimensionality 3D patch‐based undersampled reconstruction (HD‐PROST), exploiting local (within a patch), non‐local (between similar patches), and contrast redundancies.^21^ The reconstruction for the proposed technique can be formulated as the following unconstrained Lagrangian^21^

(2) $$L\left(I,T,\;Y\right)={∥EI-\sqrt{D}\;K∥}_{F}^{2}+\lambda \sum_{p}{∥T_{p}∥}_{*}+\mu \sum_{p}{∥T_{p}-P_{p}\left(I\right)-P_{p}\left(Y\right)∥}_{F}^{2},$$

where ||·||*_F_* and ||·||_*_ denote the Frobenius norm and nuclear norm respectively; *I* is the compressed T_1_ image series to be reconstructed*;* $E=\sqrt{D}AU_{r}FS$ is the encoding operator, with *S* being sensitivity maps, *F* being Fourier transform, *U_r_* being the low‐rank operator obtained by truncating the singular value decomposition (SVD) of a dictionary generated by Bloch simulation,^20^ *A* being the convolutional gridding operator, transforming Cartesian data back to 3D radial, and *D* being the non‐Cartesian density compensation function; *K* is the undersampled data; *P_p_*(·) is the patch selection operator at pixel *p* of a 3D multi‐contrast image set. This operator selects patches on local (patch for a given pixel location and contrast), non‐local (similar patches within a neighborhood for a given contrast) and contrast (patches from all the contrasts) scales and $T_{p}$ is a 3D tensor built by the selected patches centered at pixel *p*; *T* represents the denoised multi‐contrast images constructed by folding and aggregating the tensors $T_{p}$ for each pixel *p* (see the step 2 below); *Y* is the Augmented Lagrangian multiplier; *λ* is the sparsity‐promoting regularization parameter and *µ* is the penalty parameter. Equation 2 can be efficiently solved by operator‐splitting via alternating direction method of multipliers (ADMM), which divides the optimization process into the following 3 steps:

Step 1: joint reconstruction update

The first step is a joint reconstruction of the compressed TI image series (i.e., singular images) *I* by incorporating the denoised multi‐contrast images *T* obtained at the end of step 2 as prior information

(3) $$L_{\mathrm{Joint}}\left(I\right)=\begin{array}{c} \mathrm{argmin}\\ I \end{array}{∥EI-\sqrt{D}\;K∥}_{F}^{2}+\mu {∥T-I-Y∥}_{F}^{2}.$$

The above equation can be efficiently solved using the conjugate gradient (CG) algorithm.

Step 2: high‐order singular value decomposition based denoising

With obtained *I* and *Y* from step 1 and step 3 separately, the second step is to minimize the following equation regarding to the high order tensor $T_{p}$

(4) $$L_{\mathrm{Tensor}}\left(T_{p}\right)=\begin{array}{c} \mathrm{argmin}\\ T_{p} \end{array}\sum_{p}{∥T_{p}-P_{p}\left(I\right)-P_{p}\left(Y\right)∥}_{F}^{2}+\frac{\lambda }{\mu }\sum_{p}{∥T_{p}∥}_{*}.$$

The details to solve the above equation can be found in Bustin et al.^21^ Generally speaking, $T_{p}$ is obtained by thresholding the singular values obtained via high‐order singular value decomposition of the 3D tensor built by the patches selected from the multi‐contrast images. The thresholding parameter is defined by $\frac{\lambda }{\mu }$. The denoised $T_{p}$ is then rearranged to form the denoised image patches. This process is repeated for all the pixels in the singular images, and aggregation is then performed to generate the final denoised singular images *T*.

Step 3: Lagrangian multiplier update

Finally, with the optimized *I* and *T* from step 1 and step 2, the Lagrangian multiplier is updated by *Y* = *Y* + *I* − *T*. The above 3 steps are iteratively interleaved in the reconstruction process to improve the reconstructed image quality.

#### 2.3.2 Implementation details

The sequence was implemented on a 1.5 T MRI scanner (Ingenia, Philips Healthcare, The Netherlands) for cardiac imaging. The following scan parameters were used for both phantom and in vivo experiments: FOV = 200 × 200 × 200 mm^3^, spatial resolution = 1.5 × 1.5 × 1.5 mm^3^, TR/TE = 11.6 ms/5.1 ms, flip angle = 6°, IRTR = 2200 ms, number of readouts after IR = 175, *T_gap_* = 9.5 ms (minimum value allowed on the available scanner), *T_ex_* = 160.5 ms, scan duration = 9.5 min.

**Figure 3:**
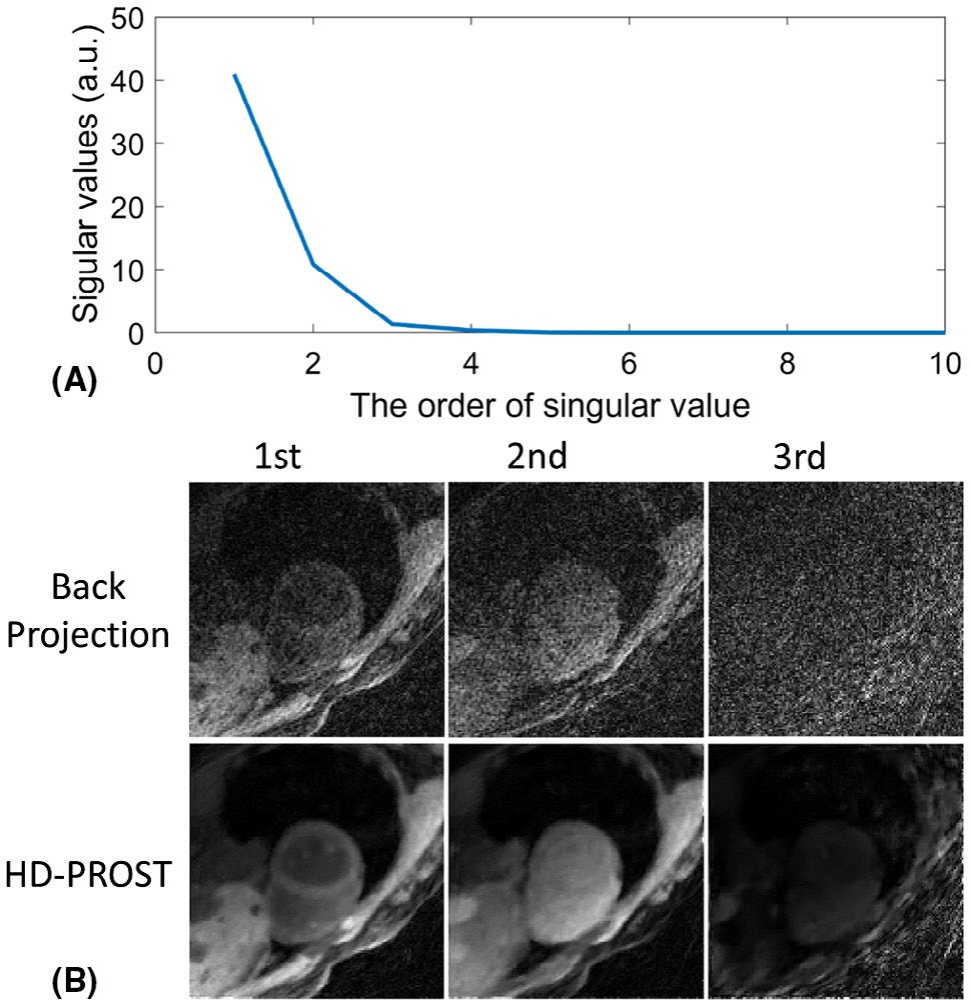
(A) The singular values obtained by singular value decomposition of the dictionary simulated according to the imaging and reconstruction parameters of the proposed 3D T_1_ mapping technique using Bloch simulation. (B) The singular value images corresponding to the 3 largest singular values in (A) reconstructed by direct back projection and HD‐PROST (high‐dimensionality 3D patch‐based undersampled reconstruction) algorithm

For the retrospective data selection for a given cardiac phase, the cardiac acquisition window was set to 186 ms, similar to the acquisition window of conventional 2D MOLLI and SASHA imaging.^10^, ^11^ The temporal window per T_1_ frame was also set to 186 ms, resulting in 10 T_1_ frames to be reconstructed. A dictionary‐based low‐rank compression^20^ was performed along the T_1_ contrast dimension to further reduce the number of T_1_ frames to be reconstructed (Equation 2). The dictionary was generated using Bloch simulation for T_1_ in the range of 100–3000 ms, with an increment of 1% with respect to the previous T_1_. The contrast of a specific TI frame is given by the average of all the radial spokes binned into the corresponding frame. The signal of each radial spoke is calculated using Bloch equation, which has been previously described for 3D radial‐based carotid quantitative mapping^24^, ^25^ and is also provided in the Supporting Information file. The singular values by SVD of the dictionary are shown in Figure 3A. The low‐rank operator *U_r_* in Equation 2 was obtained by keeping the 3 largest singular value vectors, resulting in 3 singular images to be reconstructed (Figure 3B). The first singular image, with the highest SNR, was used for patch selection in the step 2 of HD‐PROST (Equation 4).

In this study, the maximum number of CG iterations in the joint MR reconstruction (step 1) was set to 15, and the regularization parameter µ, balancing the contribution of the prior term, was empirically set to 0.01. Coil sensitivity maps were estimated by using spokes of TI larger than 1000 ms and reconstructed in low resolution by using only the central half of k‐space to reduce noise. The regularization parameter λ of HD‐PROST was optimized by visual inspection of the reconstruction quality on 1 subject and used for all other data set. For the patch‐based denoising step in HD‐PROST,^21^ the following parameters were used: patch size = 5 × 5 × 5; search window = 10 × 10 × 10; patch offset = 3; number of selected similar patches = 15. The number of ADMM iterations was set to 5. Example singular images after HD‐PROST reconstruction are shown in Figure 3B. T_1_ maps were generated by a dot product matching between the reconstructed singular images and the dictionary.

### 2.4 Phantom experiments

To test the T_1_ mapping accuracy of the 3D T_1_ mapping sequence, a standardized T_1_ phantom^26^ consisting of 9 agarose‐based vials with T_1_ values ranging from ~250–1600 ms was imaged using a 28‐channel cardiac coil. To obtain reference T_1_ values of the phantom for validation, a 2D inversion recovery spin echo (IR‐SE) sequence was performed with the following imaging parameters: FOV = 140 × 140 mm^2^, in‐plane spatial resolution = 1.5 × 1.5 mm^2^, slice thickness = 8 mm, TR/TE = 10,000/5.8 ms, 13 TIs = 50, 100, 200, 300, 400, 500, 700, 900, 1200, 1500, 2000, 2500, and 3000 ms. Standard T_1_ values were determined by a 3‐parameter non‐linear least square fitting algorithm.

For the proposed 3D radial T_1_ mapping sequence, the ECG signal used for the retrospective binning was generated by physiological simulation with heart rate varying between 50 bpm and 70 bpm during the acquisition. The acquired 3D radial data was retrospectively binned into 10 T_1_ contrasts for a simulated diastolic phase with cardiac delay of 650 ms. The phantom T_1_ map acquired with the 3D imaging sequence was generated using the HD‐PROST reconstruction as explained above.

### 2.5 In vivo experiments

The study was approved by the local institutional review board and 9 healthy subjects (5 males, 31.8 ± 3.5 y) were imaged using a 28‐channel cardiac coil. All volunteers provided written informed consent before inclusion into this study. First, standard breath‐hold 2D cine images were acquired in the mid ventricular short‐axis view with retrospective gating to 16 cardiac phases, based on which the cardiac delays for systole and diastole were determined. The T_1_ mapping reference consisted of the clinically adopted breath‐hold 2D MOLLI (3‐3‐5)^27^ and 2D SASHA^11^ sequences that were performed in diastole. The imaging parameters of MOLLI were TR/TE = 2.6/1.3 ms, flip angle = 35°, FOV = 288 × 288 mm^2^, in‐plane resolution = 2 × 2 mm^2^, and slice thickness = 8.0 mm. The SASHA sequence was performed with TR/TE = 2.6/1.3 ms, flip angle = 70°, and the same FOV and spatial resolution as used for MOLLI. Parallel imaging with SENSE acceleration factor of 2 in the phase‐encoding direction was used in the 2D MOLLI and SASHA mapping acquisitions, resulting in a mid‐diastolic acquisition window of ~187 ms. MOLLI and SASHA images were acquired in the same short‐axis location as the cine scan and reconstructed to an in‐plane resolution of 1.5 × 1.5 mm^2^. The 2D T_1_ maps for MOLLI and SASHA were obtained by fitting the corresponding standard 3‐parameter models.^10^, ^11^

The proposed free‐running 3D T_1_ mapping sequence was also acquired in short‐axis orientation covering the entire heart and centered around the slice location of the 2D MOLLI and SASHA scans. Besides diastole, T_1_ mapping was also reconstructed for systole using the proposed framework, considering that systolic T_1_ maps may find applications in patients with thin myocardium or arrhythmias.^28^, ^29^

### 2.6 Image analysis

For phantom data, circular region‐of‐interests (ROIs) were drawn for each vial and the mean and SD of T_1_ values were calculated for each vial. The accuracy of the proposed 3D T_1_ mapping technique was evaluated by comparing the estimated T_1_ values to the 2D IR‐SE method using Pearson's linear correlation and Bland‐Altman analyses.

For the analysis of in vivo images, the diastolic T_1_ map in the mid short‐axis view that was most similar to the MOLLI and SASHA geometry was selected from the 3D T_1_ maps of the proposed approach for each subject. Septal ROIs with the same size were drawn for the 3 T_1_ mapping methods. The mean and SD of T_1_ values in the septum were measured and compared between the proposed 3D method and 2D MOLLI and SASHA separately using the Wilcoxon rank‐sum test with Bonferroni correction to evaluate the in vivo accuracy and precision.

For the analysis of 3D in vivo T_1_ maps, 3 slices from base, mid to apex were selected from the diastolic and systolic T_1_ maps. T_1_ values of the myocardium were measured according to the myocardial segments model defined by the American Heart Association (AHA),^30^ with 6 segments in the base and middle slices and 4 segments in the apical slice. The mean T_1_ values were calculated for all segments to evaluate the spatial T_1_ distribution of the proposed method. The T_1_ value in each segment was compared between diastole and systole using the paired t‐test. Then the diastolic and systolic T_1_ values for each segment were determined by averaging across all the volunteers and visualized with bull's‐eye plots.

All image reconstruction and analysis were performed using MATLAB (The MathWorks, Natick, MA) on a server with a dual 16‐core CPU and 256 GB RAM. Statistical analysis was carried out in GraphPad Prism 7 (La Jolla, CA). A *P*‐value of <0.05 was considered statistically significant.

## 3 RESULTS

### 3.1 Phantom study

**Figure 4:**
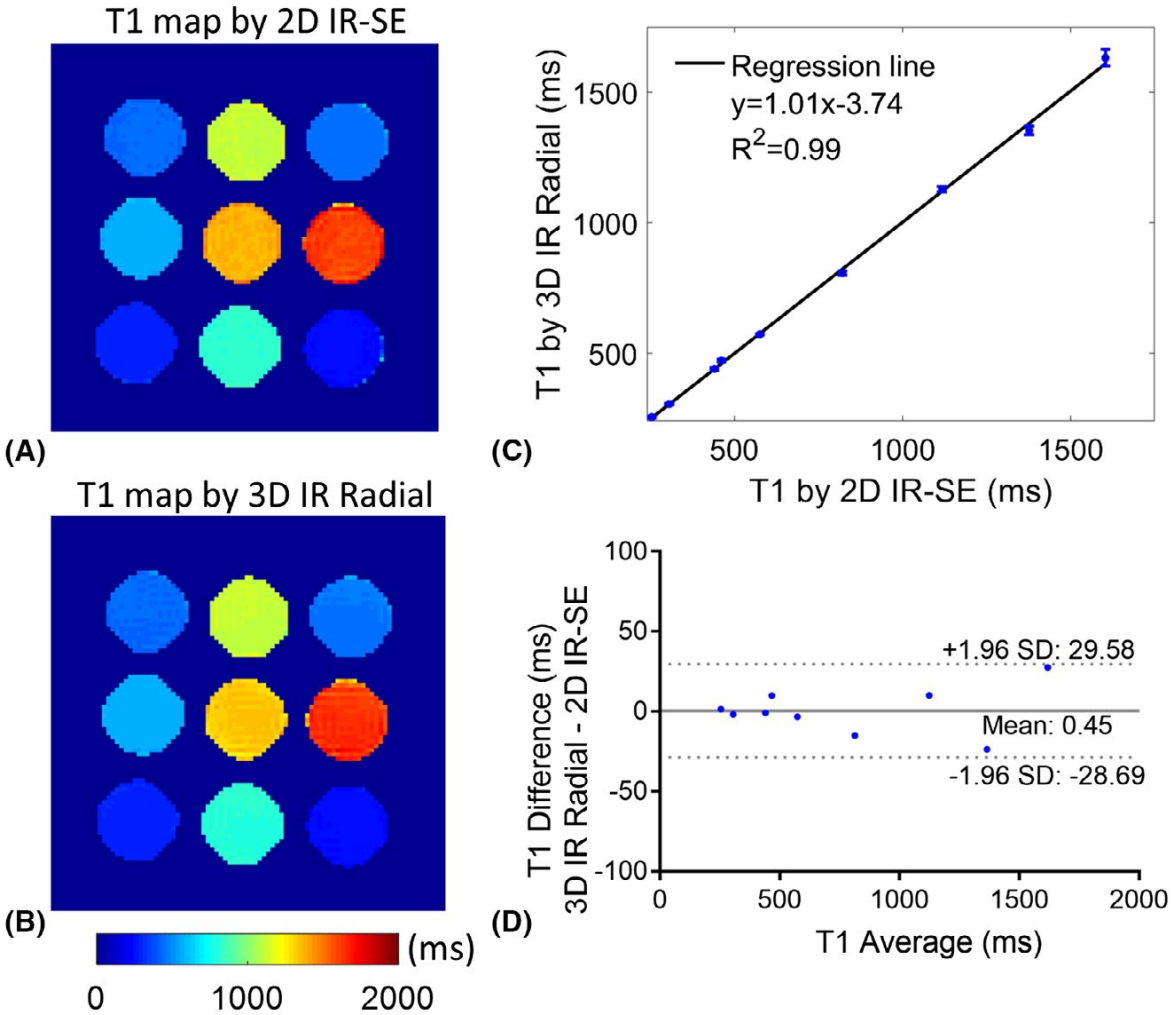
(A and B) Phantom T_1_ maps of 2D inversion recovery spin echo (2D IR‐SE) and the proposed 3D T_1_ mapping approach. (C) Linear correlation of phantom T_1_ estimation with the proposed 3D method in comparison with reference 2D IR‐SE sequence. (D) Bland‐Altman plot showing the difference between the 2 methods and their average. The grey solid line indicates the mean difference, and the grey dotted lines indicate the 95% confidence intervals of limits of agreement

Phantom T_1_ maps with the proposed approach and 2D IR‐SE are shown in Figure 4A and B, and the mean and SD of T_1_ values of all vials are shown in Figure 4C. The linear correlation indicates good agreement of T_1_ estimations between the proposed 3D technique and the 2D IR‐SE reference (R = 0.99). Bland‐Altman plot demonstrates no correlation between the difference and average of T_1_ measurements of the 2 methods (Figure 4D). Compared with 2D IR‐SE, the percentage error of the T_1_ measurements with the proposed approach was 1.2 ± 0.7%, ranging from 0.3–2.1%.

### 3.2 In vivo study

**Figure 5:**
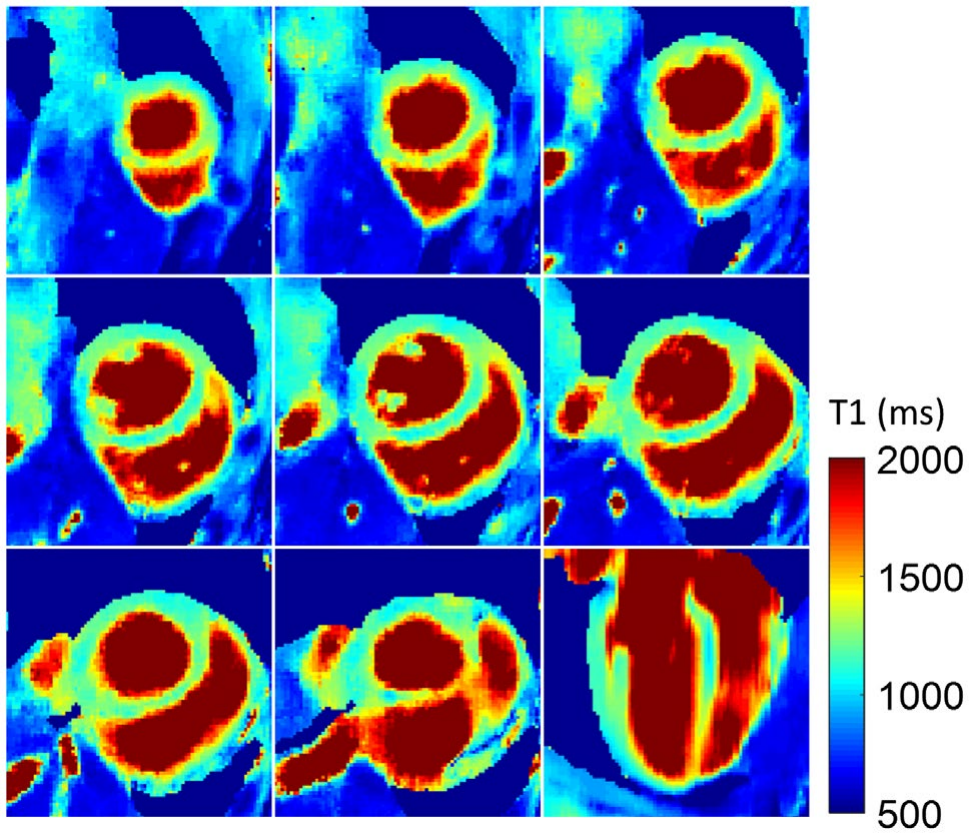
Proposed 3D T_1_ mapping technique at diastolic cardiac phase for a representative healthy subject. Eight short‐axis slices, from apex to base of the left ventricle and the reformatted long‐axis view are shown. Uniform T_1_ distribution across the myocardium can be observed

All volunteers completed the scans, and the diastolic and systolic 3D T_1_ maps were successfully reconstructed for each subject with recorded heart rates of 62 ± 8 bpm, ranging from 45–73 bpm. For reconstruction of the 3D T_1_ map, each ADMM iteration in HD‐PROST takes ~34.9 min consisting of 26.3 min for the joint reconstruction step and 8.6 min for the patch‐based denoising step, resulting in a total reconstruction time of 174.5 min with 5 ADMM iterations. T_1_ mapping results at diastole from a representative healthy subject are shown in Figure 5, consisting of 8 representative short‐axis slices and a reformatted long‐axis slice. As can be seen, the spatial distribution of myocardium T_1_ was uniform over the whole left ventricle.

**Figure 6:**
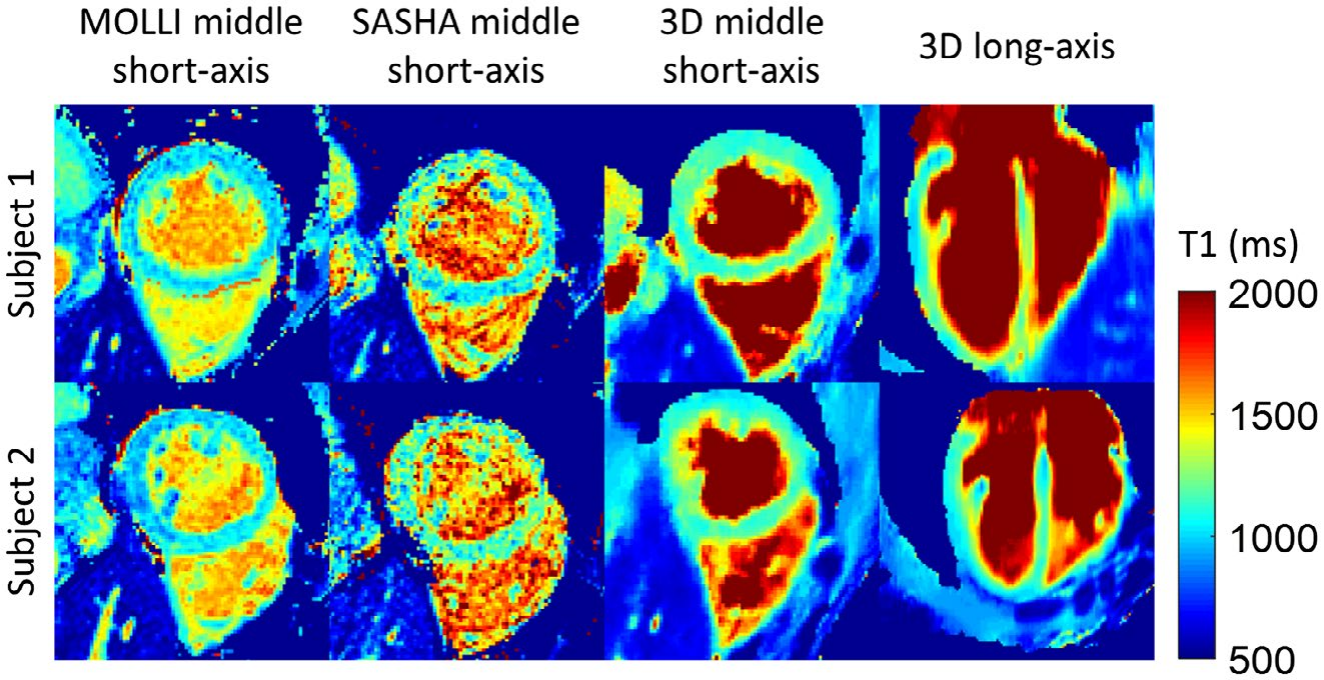
Short‐axis 2D MOLLI, 2D SASHA, and the proposed 3D T_1_ mapping results at diastolic cardiac phase for 2 healthy subjects. Long‐axis view is also included for the proposed 3D T_1_ mapping technique

**Figure 7:**
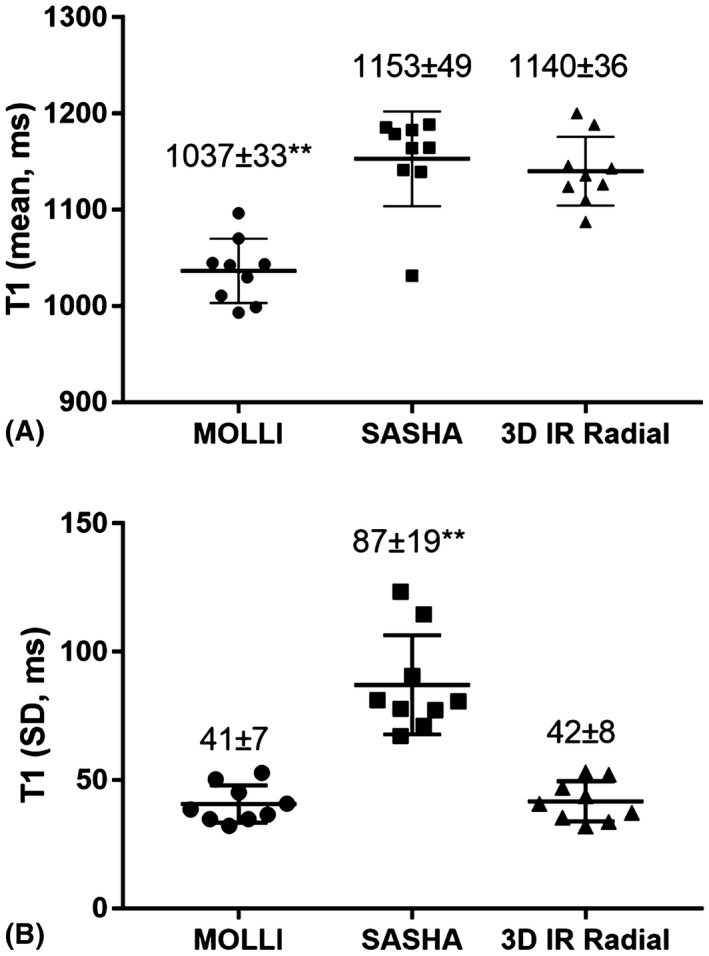
(A) Mean septum T_1_ values of all 9 healthy subjects measured with 2D MOLLI, 2D SASHA, and the proposed free‐running 3D T_1_ mapping technique. (B) SDs of the septal T_1_ measurements from all the subjects for the 3 methods. The mean ± SD across all the subjects are shown on top of each method (**P < 0.01)

Diastolic T_1_ mapping results from another 2 subjects are shown in Figure 6, including the mid short‐axis and long‐axis slices from the 3D T_1_ mapping approach and a middle short‐axis slice from 2D MOLLI and SASHA techniques. Diastolic T_1_ maps reconstructed by the proposed technique were visually comparable to the reference 2D methods with improved depiction of the right ventricle. The mean and SD of septal T_1_ derived from 2D MOLLI, 2D SASHA, and the proposed 3D technique are compared in Figure 7 for all subjects. The mean septal T_1_ measured with the proposed approach is 1140 ± 36 ms and is comparable to that of SASHA (1153 ± 49 ms) (*P *= 0.68), but is higher than that of MOLLI (1037 ± 33 ms) ( *P *< 0.01). The SD, indicating the precision of septal T_1_ measurements, was 42 ± 8 ms with the proposed approach, which is much lower than the SD of SASHA (87 ± 19 ms) (*P *< 0.01) and similar to that of MOLLI (41 ± 7 ms) (*P *= 0.79).

**Figure 8:**
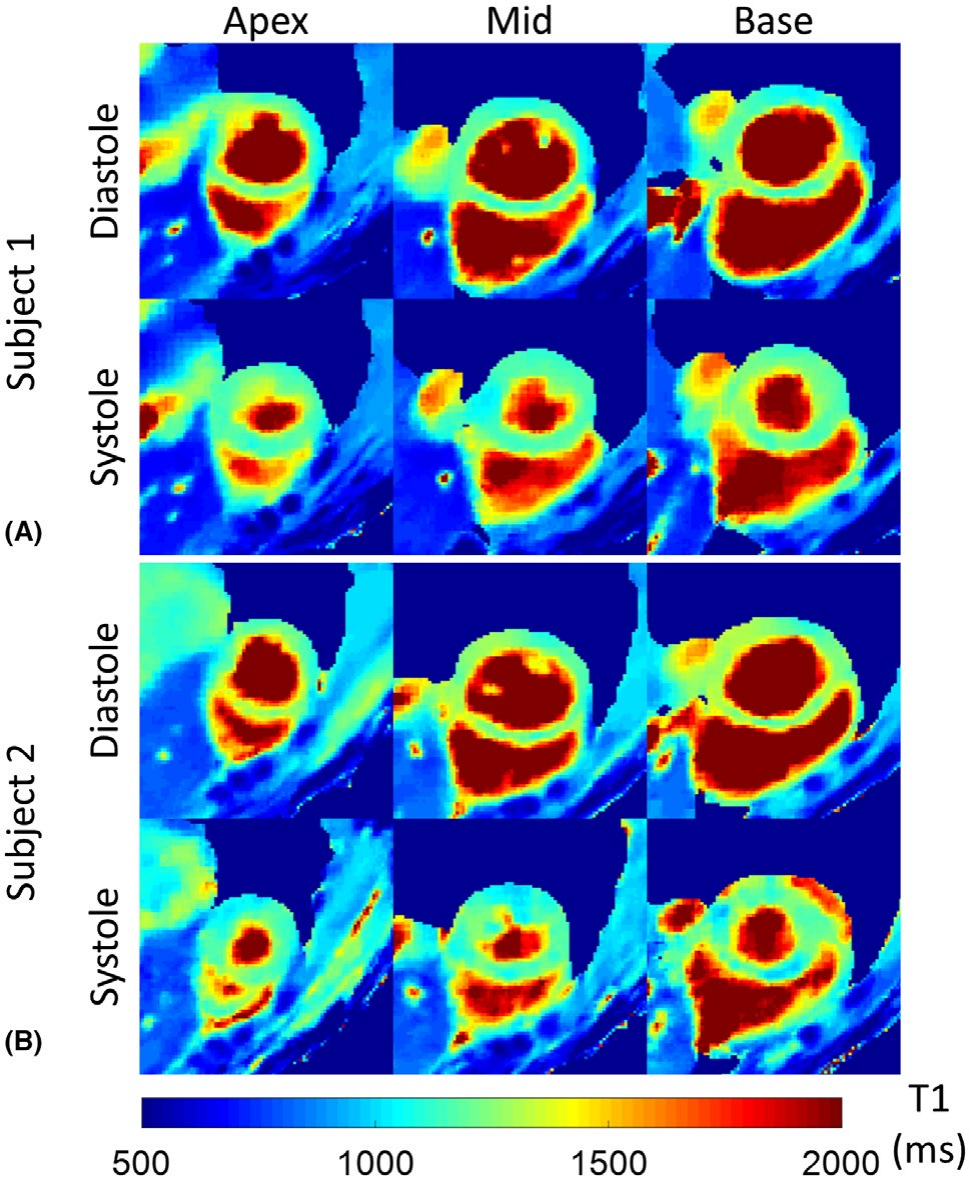
Representative diastolic and systolic T_1_ maps of basal, mid and apical short‐axis views using the proposed 3D T_1_ mapping sequence from 2 healthy subjects (A, B). Uniform myocardial T_1_ distribution can be observed on the T_1_ maps, both in diastole and systole.

**Figure 9:**
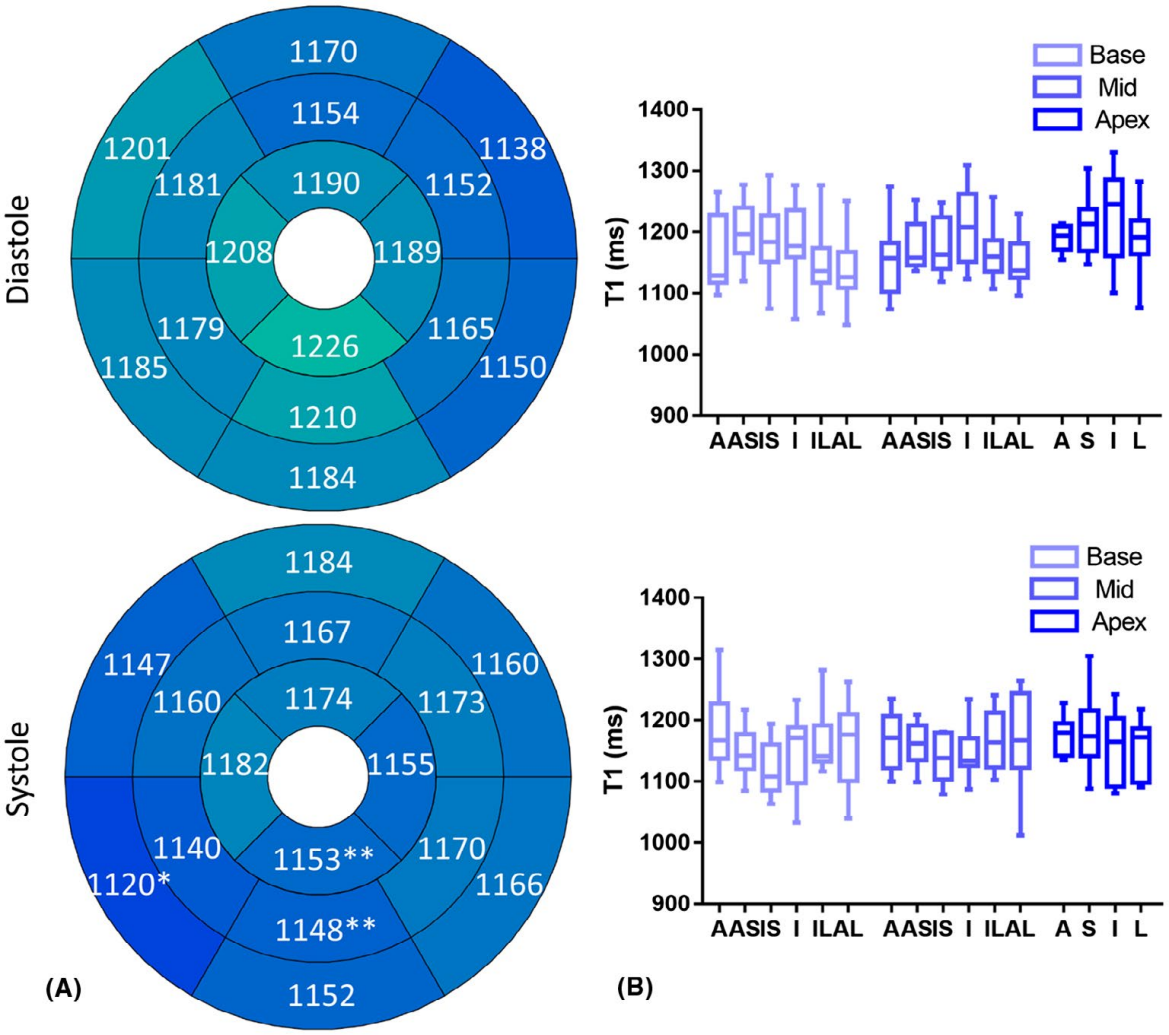
(A) AHA bull's‐eye plots showing the myocardium T_1_ distribution across the left ventricle at diastole and systole with the proposed free‐running 3D T_1_ mapping technique. The mean values obtained by averaging across all the subjects are shown in each segment (*P < 0.05, **P < 0.01). (B) Box plots showing the median, 25th, and 75th percentiles and range of the diastolic and systolic T_1_ in each AHA segment. A, anterior; AS, anteroseptal; IS, inferoseptal; I, inferior; IL, inferolateral; AL, anterolateral; S, septal; L, lateral

Representative systolic and diastolic T_1_ maps from 2 subjects are shown in Figure 8, where 3 short‐axis slices (base, mid and apex) are included. The systolic T_1_ maps also demonstrated a uniform T_1_ distribution with thicker myocardium compared with diastole. The diastolic and systolic T_1_ values of each AHA segment are shown in the bull's‐eye and box plots in Figure 9. The comparison of T_1_ values in diastole and systole for all myocardium segments are summarized in Table 1. No significant differences were found between the diastolic and systolic T_1_ values estimated with the proposed technique in most of the AHA segments, except for the inferior segments in the apical and mid ventricular slices, and the inferoseptal segment in the basal slice, with significantly longer T_1_ values in diastole than in systole.

**Table 1:** The *P*‐value of paired t‐test comparison of diastolic and systolic T_1_ values in each AHA segment with the proposed free‐running 3D T_1_ mapping technique

	A	S		I	L	
		AS	IS		IL	AL
Base	0.30	0.06	0.02*	0.10	0.57	0.73
Middle	0.57	0.25	0.13	<0.01**	>0.99	0.36
Apex	0.25	0.20		<0.01**	0.13	

Abbreviations: A, anterior; AL, anterolateral; AS, anteroseptal; I, inferior; IL, inferolateral; IS, inferoseptal; L, lateral; S, septal.

* *P *< 0.05.

** *P *< 0.01.

## 4 DISCUSSION

In this study, free‐running 3D whole heart myocardial T_1_ mapping with 1.5 mm^3^ isotropic spatial resolution has been successfully achieved with predictable 9.5 min scan time. The proposed sequence features free‐breathing, retrospective cardiac gating and continuous data acquisition, so T_1_ maps can be compensated for 3D translational respiratory motion and reconstructed at different cardiac phases by retrospective data binning. Using combined dictionary‐based low‐rank inversion^20^ and high‐dimensionality 3D patch‐based undersampled reconstruction algorithm (HD‐PROST)^21^ to reconstruct the highly undersampled 3D radial data, feasibility of myocardial T_1_ mapping was demonstrated in vivo. Myocardial T_1_ measurements with the proposed 3D technique were comparable to conventional breath‐hold, cardiac‐gated 2D MOLLI and SASHA.

In the phantom study, T_1_ values estimated with the proposed technique showed good accuracy for T_1_ ranging from 250–1600 ms as compared with the reference 2D IR‐SE. Septal T_1_ values at diastole with the proposed technique were comparable to SASHA and were significantly larger than those for MOLLI, which has been reported to underestimate T_1_.^12^ In this study, the dictionary was simulated with a linear T_1_ increment of 1%, resulting in 10–12 ms increments in the myocardial T_1_ range of 1000–1200 ms, which are small compared with the measured septal T_1_ SD (42 ± 8 ms). The precision of septal T_1_ was similar to MOLLI and significantly higher than SASHA. The results indicate that the proposed 3D T_1_ mapping technique improves accuracy compared to MOLLI and precision compared to SASHA. Therefore, the proposed technique may be a promising tool for myocardial T_1_ mapping with the advantages of both high accuracy and precision. In addition, with the proposed 3D high resolution T_1_ mapping technique, visualization of the right ventricle was improved, which may facilitate fibrosis quantification in the thin wall of the right ventricle. Nevertheless, T_1_ values of the thin right ventricle should be interpreted carefully, considering that there may be partial volume effects resulting from residual fat caused by the imperfection of the water selective excitation pulse.

In this study, respiratory motion was addressed by correcting the phase of k‐space data using motion parameters estimated from 3D translational image registration of images acquired at different respiratory states. More sophisticated registration methods, such as affine and non‐linear transforms could be considered in future work to account for more complex respiratory‐induced cardiac movement. To extract reliable cardiac trigger signal to account for cardiac motion, ECG signal was logged and synchronized with data acquisition for retrospective data selection. Previous studies have proposed several approaches for cardiac self‐navigation using radial or spiral trajectories.^22^, ^23^ Therefore, cardiac self‐navigation, besides respiratory self‐navigation, could be also investigated in future studies to achieve free‐breathing, ECG‐free 3D myocardial T_1_ mapping.

T_1_ mapping at systole has special diagnostic value when the diastolic T_1_ map cannot be reliably obtained, such as in patients with thin myocardium, where partial volume effects may corrupt myocardial T_1_ estimation, and in patients suffering from frequent arrhythmias, where the diastolic onset and duration are changing.^28^, ^29^ In this study, systolic T_1_ maps were reconstructed along with diastolic T_1_ maps for each subject. The AHA segment analysis showed good spatial uniformity of the myocardial T_1_ values measured across the left ventricle in both diastole and systole. In general, the systolic myocardium T_1_ was comparable to the diastolic myocardium T_1_. The shorter T_1_ in the inferoseptal segment of the basal slice and inferior segments of the mid and apical slices may be explained by increased myocardial thickness and therefore reduced partial volume effects from blood during systole compared to diastole.^29^ The acquisition window for T_1_ mapping was set to ~186 ms, which was adequate for diastole, but may not be optimal for systole. In spite of this, radial data acquisition is less sensitive to motion than Cartesian sampling, and no obvious motion artefacts were observed in the systolic T_1_ maps.

Systolic and diastolic T_1_ maps were reconstructed for each subject separately in our experiments. Instead of separate reconstruction for each cardiac phase, cardiac‐resolved T_1_ maps can be reconstructed simultaneously, by which additional redundancy along cardiac phases can be exploited and improved reconstruction performance can be expected at the expenses of memory requirement and computational times. Furthermore, 3D cardiac cine reconstruction from the free‐running sequence should be also possible. This could be done by sorting the respiration motion compensated k‐space data with a small temporal window along the cardiac phase (e.g., 50 ms) and a large temporal window along contrast. After binning, the undersampled k‐space data of different cardiac phases could be reconstructed simultaneously, e.g., by adding a total variation constraint along the cardiac phase direction^31^ to the reconstruction Equation 2. This approach will be investigated in future studies.

SPGR readout with low flip angle instead of balanced steady‐state free precession (bSSFP) readout was used in the proposed sequence. Although bSSFP readout may have SNR benefits, the low flip angle SPGR readout is less sensitive to B_1_ and B_0_ field inhomogeneities that will influence T_1_ estimation and may cause banding artefacts in the image. The proposed technique was investigated at 1.5 T in this study, but has potential to be extended to 3 T. Furthermore, benefits of higher SNR, and therefore shortened scan time or improved spatial resolution can be expected at 3 T.

Several limitations of the proposed technique need to be mentioned. First, the proposed T_1_ mapping technique cannot estimate T_1_ of flowing blood accurately. This is because the signal model used for T_1_ mapping assumes that the tissue of interest is static and experiences all the inversion and excitation pulses in the sequence, which may not be true for flowing blood. When measurement of myocardial extracellular volume is needed, estimation of blood T_1_ could be performed using a free‐breathing rapid low spatial resolution (enough to only define a region of interest within the blood pool) T_1_ mapping sequence acquired in a mid‐ventricular slice. Second, in this study the proposed technique was only tested for native myocardial T_1_ mapping. However, post‐contrast myocardial T_1_ mapping using this technique should be also feasible. Furthermore, considering the reduced T_1_ after contrast injection, shorter IRTR could be adopted and shorter scan duration can be expected. Third, in the free‐running sequence with retrospective binning, although inter‐bin respiratory and cardiac motion has been carefully addressed, compared with breath‐hold images, there may still be some remaining intra‐bin respiratory motion and cardiac motion that result in some blurring of the reconstructed images. Future studies will investigate the performance of the proposed sequence in a cohort of patients with cardiovascular disease. Last, the current implementation of HD‐PROST reconstruction is suboptimal and it takes ~3 h to reconstruct a whole heart 3D T_1_ map. GPU implementation will be investigated in the future for the non‐uniform Fourier Transform^32^ and the patch‐based denoising process to accelerate the reconstruction.

## 5 CONCLUSIONS

In this study, a free‐running 3D myocardial T_1_ mapping technique with whole heart coverage and high isotropic spatial resolution is proposed. 3D T_1_ mapping was shown to have good accuracy and precision in comparison to reference sequence in phantom and conventional T_1_ mapping methods in in vivo experiments. Based on retrospective data selection and combined dictionary‐based low‐rank inversion and patch‐based reconstruction, high resolution 3D T_1_ maps could be obtained for different cardiac phases. Future studies will investigate the clinical value of the proposed technique.
