# Supplementary material for: Free‐running 3D whole heart myocardial T1 mapping with isotropic spatial resolution
Source: Magn Reson Med. 2019 May 17;82(4):1331–42. doi: 10.1002/mrm.27811 (PMC6851769; doi:10.1002/mrm.27811)
Supplement: Supplementary file 1 [file MRM-82-1331-s001.docx]

**Supporting Information**

**Signal Equation for the Proposed 3D T1 Mapping Technique**

Assuming the longitudinal magnetization of static tissue before the $m^{th}$ IR pulse is $M_{IR}^{m}$, and $M_{IR}^{1}=M_{0}$, according to Bloch equation the magnetization after the $m^{th}$ IR pulse and before the first $\theta$ pulse is given by:

$M_{z}^{m}\left( T_{gap}^{-} \right)=M_{0}-(M_{0}+M_{IR}^{m})E_{gap}$ [S1]

where the relaxation term is given by $E_{gap}=exp(-T_{gap}/T_{1})$. $T_{gap}$ is the gap between the IR pulse and the first excitation and $T_{ex}$ is the time between the last excitation in the readout and the next IR pulse. The signal right before the $k^{th}$ ($k$>1) $\theta$ pulse is:

$M_{z}^{m}\left( k_{\theta}^{-} \right)=M_{z}^{m}\left( T_{gap}^{-} \right){(E_{1}cos\theta)}^{k-1}+M_{0}(1-E_{1})\frac{1-{(E_{1}cos\theta)}^{k-1}}{1-E_{1}cos\theta}$ [S2]

where $E_{1}=exp(-TR/T_{1})$ and $TR$ is the repetition time of the $\theta$ pulse. Then, the signal right before the next IR pulse is given by,

$M_{IR}^{m+1}=M_{0}\left( 1-E_{e} \right)+M_{z}^{m}\left( {(N+1)}_{\theta}^{-} \right)E_{e}$ [S3]

where $E_{e}=exp(-T_{ex}/T_{1})$, $N$ indicates the number of readouts within each IRTR. Using Eq. [S1-S3], the signal acquired by each spoke can be simulated for a given T1.
